# Multistate Competing Risk Analysis of Transition Back to the Community Among Long-Term Care Home (LTC) Destined Patients: A Brief Report

**DOI:** 10.1177/21501319231220742

**Published:** 2023-12-22

**Authors:** Bonaventure A. Egbujie, Jake Tran, John P. Hirdes

**Affiliations:** 1 University of Waterloo, Waterloo, ON, Canada; 2 Toronto Grace Health Center, Toronto, ON, Canada

**Keywords:** geriatrics, long term care, interRAI, aging, multistatate transition

## Abstract

**Objective:**

The demand for long-term care in community and facilitybased settings in Canada is expected to increase with population growth. The Toronto Grace Health Center piloted an intervention program that aims to support return to the community of acute hospital patients designated for LTC placement. We investigated whether this program was effective in transitioning the program patients back to their homes in the community and the factors associated with transitioning patients to different destinations.

**Method:**

We performed a competing risk multi-state analysis of 111 patients enrolled into the Harbour Light (HL) transitional unit program between January 2020 and June 2023.

**Results:**

At the time of the study, 92 enrolled patients had been discharged and of those these, 48.9% (45) were successfully transitioned back to their private home in the community. The remaining 51.1% (46) were discharged to other destinations. Being a female was the only positive predictor of transitioning back home. Higher CPS scores (HR 0.53, 95% CI 0.31-0.88), PADDRS scale of 1+, and higher ADL Hierarchy scale, strongly predicted lower odds of transitioning back to the community.

**Conclusion:**

Within the context of rising LTC bed demand and lengthy waiting time in Canada, with appropriate measures, this program successfully transitioned LTC home bound persons back to their homes. If replicable on a large scale, this could provide short and long-term solution to LTC bed demand in Canada.

## Introduction

Population aging is often accompanied with a growing demand for long-term care (LTC) homes as the immediate solution to caring for persons with complex needs.^1^ The demand for long-term care in community and facility-based settings in Canada is expected to increase with population growth.^1-3^ The cost associated with dramatic expansion of facility-based care could be prohibitive and the preferences of most older adults is to remain at home for as long as possible.^1^ Hence, short- and long-term solutions are required.

With the supply of LTC beds not keeping pace with levels of demand, finding new ways to reduce LTC bed demand may be a more practical short-term.^2^ One such strategy may involve increasing the number of people transitioned back to the community following admission for acute care in hospitals rather than to LTC homes. However, Nord^4^ suggests such transitions can be challenging and could result in delayed discharge from acute care (also referred to as Alternate Level of Care [ALC] days). Transitions back to the community can occur when persons are placed in long-term care homes,^5^ however, these tend to occur within the first 90-days of stay for a very small portion of persons.

Arthur et al^6^ showed that home care clients admitted to acute care were more likely to experience delayed discharge with advanced age, social vulnerability, functional impairment, behavior problems, Parkinsonism, and dementia.^6^ Costa et al^7^ reported that among acute care hospital patients with delayed discharge, 23% of patient days were for 4% of patients with morbid obesity, psychiatric diagnoses, abusive behaviors, and stroke. This suggests that interventions targeting these specific subsets of patients may be useful.

Transitional care units were established in Ontario to address some of the challenges associated with delayed discharge from acute care hospitals^4^ and emerging reports suggest some success with the strategy.^8^ Such transitional units could also be utilized as a vehicle through which patients are transitioned back to the communities, especially if appropriate person-centered measures are taken.

The Toronto Grace Health Center set up the Harbour Light (HL) transitional care program that aims to support return to the community of acute care hospital patients already designated for LTC placement. The strategic approach of the HL program is to discharge patients to a supportive clinical environment from the acute care hospital for additional intervention (see below) with the aim of preventing LTC facility placement.

We investigated whether this program was effective in transitioning enrollees back to their homes in the community and what factors enable or hinder such transition.

## Methods

### Study Design

This involved a longitudinal analysis of patients enrolled into the Harbour light (HL) transitional unit program between January 2020 and June 2023 using competing risk multistate design.^5,9-12^

**Figure 1.**
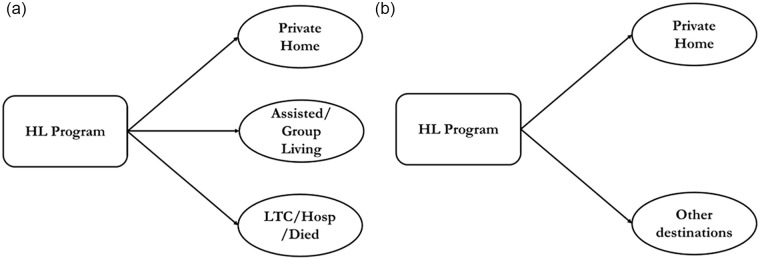
State-space d﻿iagram for the c﻿ompeting r﻿isk m﻿odel s﻿howing the a﻿pproach used for (a) Descriptive, and (b) Inferential Analyses.

Multistate competing risk approach is appropriate when an event history analysis is desired, and the study subjects could transition to several mutually exclusive states (absorbing states) as the terminal outcome.^11,13,14^ In the HL program, discharge destinations are mutually exclusive, with discharge to 1 destination precluding discharge to any other destination.^13^ A state space diagram for this study is presented below (Figure 1).

### Source Data

We analyzed data obtained from the routine assessments of patients who were enrolled into the HL program. Patients were assessed using the interRAI PAC-Rehab instrument^15-18^ and all assessments were completed by trained assessors immediately on admission or as soon as possible. This assessment is then repeated every 90 days, and a final assessment is performed at the time of the patient’s discharge. The interRAI PAC-Rehab instrument is designed for use in post-acute care settings as part of interRAI’s suite of hospital assessment systems.^19-23^

### Intervention

HL program utilizes integrated and coordinated services from community providers, “Reconnect” for mental health & addiction services and “The Salvation Army-Toronto Grace” for instrumental and basic activities of daily living (IADL and ADL) related services. This enables safe transitioning of post-bariatrics and Mental Health & Addiction patients with a current long-term care placement application to an alternative permanent place of living. ALC patients with post-bariatric or MH&A needs as barriers to placement are identified from different acute care hospitals within Toronto area and enrolled into the HL program. Before enrollment, a care navigator identifies the support needs of patients and collaboratively defines their care goals.

On enrollment, a long-term transition plan is developed between TGHC/HL, Reconnect, and community support teams, while the patient receives care at the HL unit. Thereafter, transitional Services that include Occupational or physical therapy, personal support services, nursing care, or other services are provided up to 90 days (MH&A) or 180 days (post-bariatric) in duration for the patients. At the end of the transitional care duration, the patients are discharged to the community (or other settings) and continue to receive ongoing follow-up and care.

### Outcome of Interest

The primary outcome of interest was the transition destinations of HL patients, which we classified as discharge to “*Private home*” versus “*Other settings.*” The PAC-R differentiates between 17 discharge destinations, but for descriptive analysis we initially collapsed the 17 items into 3 groups namely, 1—Private home, 2—Assisted/Group Living, and 3—LTC/Hosp/Died. Supplemental File 1 shows the possible 17 discharge destinations and how they are grouped into the above categories. For all multistate competing risks analysis, the outcome of interest was modelled as 3 states namely: “0—Censored,” “1—Private home,” and “2—Other destinations.” Patients were censored if they are still on admission at the cutoff date when data were extracted for this analysis . Both “Private home” and “Other destinations” are *absorbing states* meaning that a discharge to 1 of them precludes discharge to the other.

### Independent Variables

We selected a mixture of patient-level and process variables that include socio-demographic factors (eg, sex and age), clinical factors (eg, fall within 3 days of admission, depression, stroke, heart failure, and self-rated health) as predictors. Other variables considered include clinical summary scales such as the Aggressive Behavior Scale^24^ (ABS), Cognitive Performance Scale^25^ (CPS), interRAI ADL hierarchy scale^26^ (ADLH) which is a 6-category composite variable obtained by scoring 4 ADL items (locomotion, eating, toilet, and personal hygiene), the Post-Acute Delayed Discharge Risk Scale^27^ (PADDRS) were also used. PADDRS is a clinical decision tool that combines numerous important clinical factors associated with delayed discharge from a post-acute hospital.

### Statistical Analysis

A descriptive analysis of the characteristics of study participants was performed using frequencies and percentages for categorical variables or mean and standard deviations (median and interquartile range, IQR where there are outliers) for continuous variables. We further performed bivariate analysis to assess relationships between the dependent and independent variables. To measure the strength of association between 2 categorical variables we used Fisher’s exact test because of the small size of our dataset, which caused our contingency table to have expected frequency less than 5 in multiple cells. Kruskal Wallis test was used to check for differences in the median values of continuous variables between the discharge destination groups, having established their non-normal distribution. We plotted the cumulative incidence function (CIF) graph to display the cumulative intensities of transitioning to the different destinations.

Multistate competing risk cox regression model was used to perform inferential analysis.^28^ First, we performed univariate cox regression analysis to check for unadjusted risk of transitioning back to home for the selected independent variables. Variables found to be associated with risk of transition to any destination were included in the multivariable model.

We then fit a multivariable multistate competing risk model to the data, to determine each variable’s independent association with the risk of transitioning to “Private home” or “Other destinations,” accounting for the simultaneous effect of other variables. We fit the multistate model using the “*coxph*” function of *Survival* package in R statistical software. All analyses were performed in R statistical software version 4.4.2 (2022-10-31 ucrt)—“Innocent and Trusting.” Copyright© 2022. The R Foundation for Statistical Computing.

## Results

### Characteristic of HL Patients

One hundred and eleven (111) unique patients with complete admission information were included in this analysis. At the time of the study, 92 of the 111 had been discharged and of those discharged, 48.9% (45) were successfully transitioned back to their private home in the community. The remaining 51.1% (46) were discharged to other destinations during the period of admission. The mean age of discharged patients was 57.9 years (SD = 15.0), and 63.0% were below 65 years, 36.9% (33) were females. Table 1 shows the admission characteristics of the 92 patients already discharged from the program.

**Table 1.** Baseline Characteristics of Harbour Light Enrolees by Their Discharge Destination.

Variables	Category	Total n = 92	Private home n = 45 (49%)	Other n = 46 (51%)	*P* values (Fischer’s exact)
		n (%)	n (%)	n (%)	
Age group (years)	<65	58 (63.0)	26 (57.8)	32 (68.1)	.39
	≥65	34 (37.0)	19 (42.2)	15 (31.9)	
Sex	F	33 (36.9)	21(46.7)	12 (25.5)	.05
	M	59 (63.1)	24 (53.3)	35 (74.5)	
ADL Hierarchy Scale	0	83 (91.2)	41 (93.2)	42 (89.4)	.72
	1+	8 (8.8)	3 (6.8)	5 (10.6)	
ABS Scale	None	79 (86.8)	40 (90.9)	39 (83.0)	.36
	Mild-severe	11 (13.2)	4 (9.1)	8 (17.0)	
Median LOS (days)		77	77	90	.26
PADDRS	0	68 (73.9)	39 (86.7)	31 (66.6)	.29
	1+	24 (26.1)	6 (13.3)	16 (33.4)	
Stroke	No	83 (91.2)	40 (90.9)	43 (91.5)	1.0
	Yes	8 (8.8)	4 (9.1)	4 (8.5)	
Usual home available	No	25 (64.1)	7 (41.2)	18 (81.8)	.02
	Yes	14 (35.9)	10 (58.8)	4 (18.2)	
DRS	<3	53 (58.2)	22 (50.0)	31 (66.0)	.14
	3+	38 (41.8)	22 (50.0)	16 (34.0)	
Unsteady gait	No	58 (63.7)	26 (59.1)	32 (68.1)	.39
	Yes	33 (36.3)	18 (40.9)	15 (31.9)	
Falls current	No	84 (92.3)	41 (93.2)	43 (91.5)	1.0
	Yes	7 (7.7)	3 (6.8)	4 (8.5)	

### Cumulative Incidence of Transition to Various Destinations

**Figure 2.**
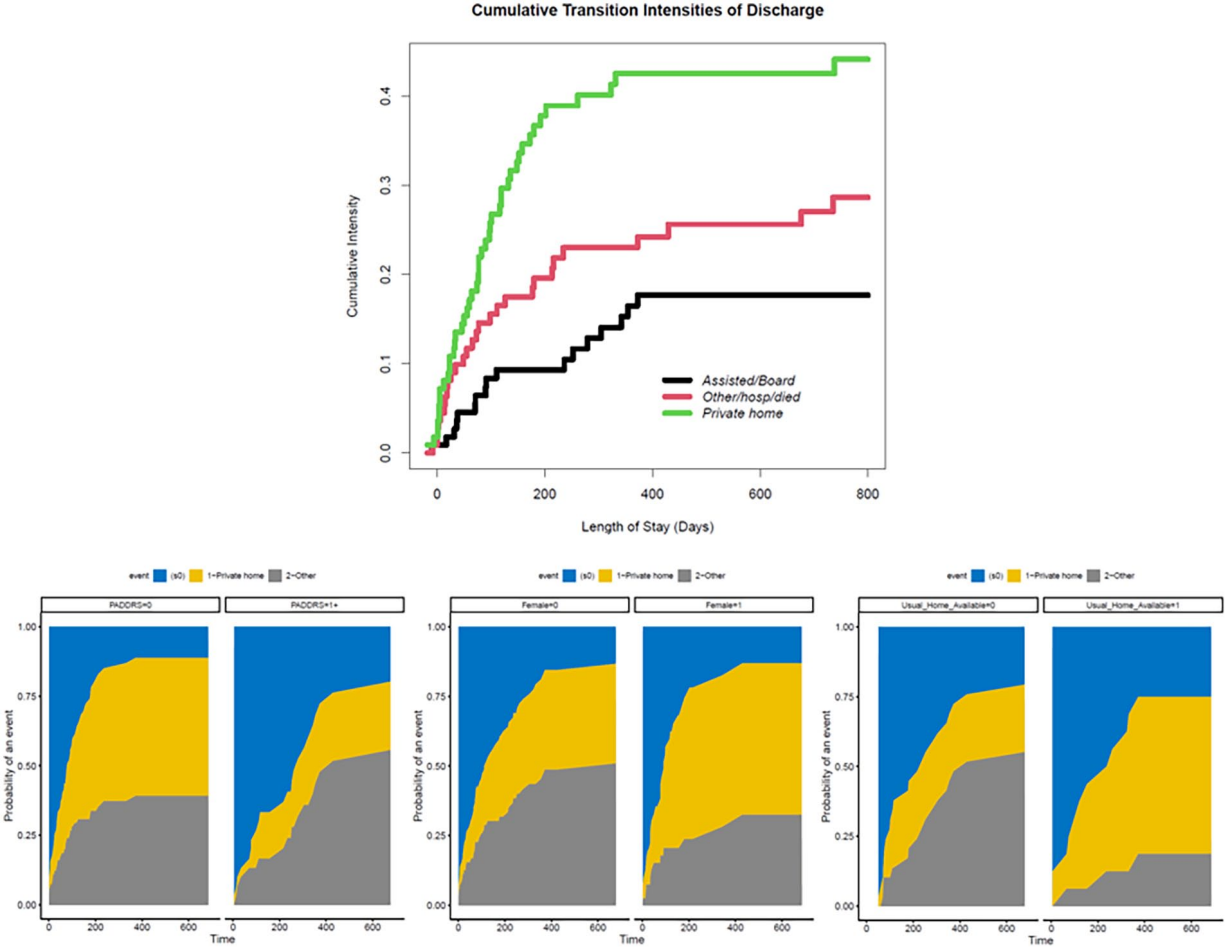
Cumulative Incidence (CIF) Plot Showing The Cumulative Incidence Of Transition to Different Discharge Destinations generally (upper), Stratified by Various Variables (lower).

The distribution of destinations for discharged HL program patients were Private home (45, 48.9%), Other (10, 10.9%), Assisted living (8, 8.7%), Board and care (7, 7.6%), Acute care hospital (5, 5.4%), Homeless (4, 4.3%), Residential care[includes LTC home] (3, 3.3%) Mental health Residence (2, 2.2%), Psychiatric unit (2, 2.2%), Group home for people with physical disability (2, 2.2%), Continuing care facility (2, 2.2%), Home for people with intellectual disability (1, 1.1%), and Rehabilitation unit (1, 1.1%). The CIF plot shows an early and steep rise in transition intensity (TI) to private homes with somewhat later and equally steep rise in TI to other destinations (*see methods section for description of other destinations*), Figure 2. The plot showed that the cumulative TI to other destinations continued to rise later on even when TI to private home has plateaued. Figure 2 also shows the CIF plot of transitioning to different destinations according to patient-level risk factors.

### Unadjusted Hazard Ratio of Discharge to Different Destinations

Table 2 shows the results of univariate cox regression analyses showing unadjusted risk of discharge to different destinations. Having the usual home available (HR 2.61, 95% CI 1.03-6.60) and being a female (HR 1.85, 95% CI 1.04-3.27) were positively associated with successful discharge back to the community. Higher CPS scale scores (HR0.57, 95% CI 0.38-0.85), and an admission PADDRS scale of 1+ (HR 0.25, 95% CI 0.11-0.58) were strong predictors of lower odds of successful transition back to the community, Table 2. Experiencing a fall around the time of admission into the program (HR 2.44, 95% CI 1.05-5.65) strongly predicted more likelihood of discharge to other destinations.

**Table 2.** Unadjusted Odds of Transitioning to Home vs Other Destination Among Harbour Light Enrolees.

Variables	Category	Private home	Other destinations	*P*-value
		HR (95% CI)	HR (95% CI)	
Age group (years)				
	<65	Ref		.30
	≥65	1.11 (0.63-1.98)	0.65 (0.36-1.17)	
Sex	M	Ref		.10
	F	1.93 (1.05-3.55)	0.49 (0.10-2.33)	
ADL Hierarchy Scale	0	Ref		.04
	1+	0.34 (0.18-0.98)	0.53 (0.22-1.25)	
ABS Scale	None	Ref		.40
	Mild-severe	0.52 (0.23-1.20)	1.18 (0.55-2.54)	
PADDRS	0	Ref		.0006
	1+	0.25 (0.11-0.58)	0.67 (037-1.21)	
Stroke	Yes	1.20 (0.47-3.06)	1.22 (0.40-3.77)	.90
Unsteady gait	Yes	1.45 (0.79-2.64)	1.13 (0.59-2.20)	.50
Usual home avail	Yes	2.61 (1.03-6.60)	0.47 (0.18-1.28)	.05
Depression	Yes	2.15 (1.18-3.91)	1.39 (0.77-2.51)	.03
Falls current	Yes	1.60 (0.52-4.90)	2.44 (1.05-5.65)	.3

### Multivariable Cox Regression Analysis

**Figure 3.**
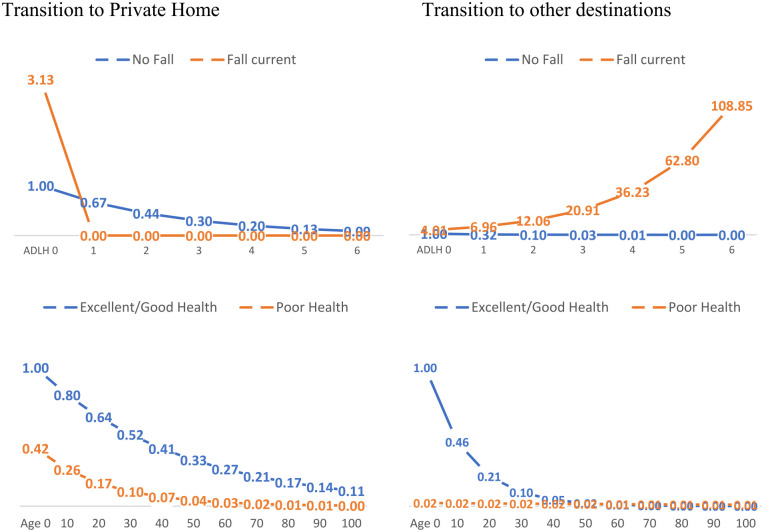
Interaction Term Showing the Adjusted Odds of Transitioning Back Home vs. Other Destination upper) Fall and ADL Hierarchy, lower) Self-rate health and Age.

In the final multivariable cox regression analysis, being a female was the only positive predictor of transitioning back home (HR 2.51, 95% CI 1.26-4.99). Higher CPS scores (HR 0.53, 95% CI 0.31-0.88), PADDRS scale of 1+, and higher ADL Hierarchy scale, strongly predicted lower odds of transitioning back to the community, while heart failure (HR 2.01, 95% CI 1.34-2.99), fall around the time of admission, is associated with greater odds of transitioning to destinations other than home. A complete list of all 9 variables included in the model and their hazard rates are presented in Table 3. We accounted for interactions between patient factors by including their interaction terms in the final model. Having a fall around the time of admission into the HL program showed strong significant interaction with ADL Hierarchy scale, as well as with PADDRS score. Those who had a fall around the time of admission “additionally” were significantly less likely to transition back to their private homes as their ADL hierarchy scale increases (Figure 3). Patients with fair or poor self-rated health were significantly less likely to transition back home or to other destinations, and as age increases, the likelihood of transitioning out of the program back home or to other destinations further diminishes, more so for patients with fair or poor health (Figure 3).

**Table 3.** Adjusted Odds of Transitioning to Home vs Other Destination Among Harbour Light Enrolees.

Variable	Category	Private home vs being censored	Other destinations vs being censored
		HR (95% CI)	HR (95% CI)
Age		0.98 (0.95-1.01)	0.93 (0.88-0.97)
Female		2.51 (1.26-4.99)	1.12 (0.53-2.38)
ADL Hierarchy Scale		0.67 (0.29-1.53)	0.32 (0.13-0.77)
CPS Scale		0.53 (0.32-0.88)	0.89 (0.57-1.38)
ABS Scale		0.79 (0.49-1.27)	1.32 (1.06-1.64)
PADDRS	0	Ref	
	1+	0.22 (0.09-0.49)	0.45 (023-0.88)
Fall current	Yes	3.12 (0.91-10.66)	4.01 (1.39-11.55)
CHF	Yes	1.73 (0.73-4.10)	2.00 (1.34-2.99)
Health status	Excellent/good	Ref	
	Fair/poor	0.42 (0.03-5.29)	0.03 (0.0009-0.68)

Overall, our final multivariable model contained 9 covariates, 2 interaction terms and achieved a concordance index of 0.77 with a likelihood ratio of *P*-value, .0000003.

Using the global test, assumption of proportionality in the model was not violated with a *P*-value >.05. Supplemental File 2 shows the full result of the proportional hazard assumption check.

## Discussion

Our analyses show that a substantial proportion of patients enrolled in the HL program successfully transition back to their private homes in the community. Only 3 enrolled patients transitioned to residential care (equivalent of LTC). Being cognitively and physically impaired, having a higher score on PADDRS scale, at the time of enrollment, strongly predicted less likelihood of successful transition back to the community. Consequently, having multiples of these factors exponentially reduced the chances of community transition.

Results of our analysis suggest that the HL program attained its primary objective which was to transition patients back to their homes instead. It is worth re-emphasizing that all the patients enrolled into the HL program were previously destined for LTC homes but were on the waitlist.

The analysis also shows that where successful transition back to private homes occurred, the transition happened rapidly in early stages of the program. This suggested that there is little extra gain made keeping patients very long in the program if the target is to successfully transition them back to the community.

A risk stratification tool could be developed from the delineated predictors of successful and unsuccessful home transition among this study’s participants. Such a tool can be based on interRAI assessment data and can be used to identify LTC-bound patients who could successfully transition back to the community with the right types and intensity of support.

### Limitations

A major limitation of our study is the small sample size, which may affect the power of our models to detect changes in the outcomes of interest. We are therefore cautious in interpreting the effect sizes obtained from this study, especially where the confidence interval is very wide. We also note that patients in this cohort had a mean age of <60 years, which is not typical of LTC-bound patients.

## Conclusions

Within the context of rising LTC bed demand and lengthy waiting time in Canada, the success of the program, if replicable (with a larger sample), could offer new solution to a growing health resource challenge.

Further analysis with a larger sample size will be necessary to increase the granularity and precision of findings. It will also be worth following up the patients who transitioned back to their homes to determine the duration of their stays in the community.

## Supplemental Material

sj-docx-1-jpc-10.1177_21501319231220742 – Supplemental material for Multistate Competing Risk Analysis of Transition Back to the Community Among Long-Term Care Home (LTC) Destined Patients: A Brief Report Click here for additional data file. Supplemental material, sj-docx-1-jpc-10.1177_21501319231220742 for Multistate Competing Risk Analysis of Transition Back to the Community Among Long-Term Care Home (LTC) Destined Patients: A Brief Report by Bonaventure A. Egbujie, Jake Tran and John P. Hirdes in Journal of Primary Care & Community Health

sj-docx-2-jpc-10.1177_21501319231220742 – Supplemental material for Multistate Competing Risk Analysis of Transition Back to the Community Among Long-Term Care Home (LTC) Destined Patients: A Brief Report Click here for additional data file. Supplemental material, sj-docx-2-jpc-10.1177_21501319231220742 for Multistate Competing Risk Analysis of Transition Back to the Community Among Long-Term Care Home (LTC) Destined Patients: A Brief Report by Bonaventure A. Egbujie, Jake Tran and John P. Hirdes in Journal of Primary Care & Community Health
