# Supplementary material for: Multistate Competing Risk Analysis of Transition Back to the Community Among Long-Term Care Home (LTC) Destined Patients: A Brief Report
Source: J Prim Care Community Health. 2023 Dec 22;14:21501319231220742. doi: 10.1177/21501319231220742 (PMC10748573; doi:10.1177/21501319231220742)
Supplement: sj-docx-1-jpc-10.1177_21501319231220742 – Supplemental material for Multistate Competing Risk Analysis of Transition Back to the Community Among Long-Term Care Home (LTC) Destined Patients: A Brief Report [file sj-docx-1-jpc-10.1177_21501319231220742.docx]

|  | **Discharge Destination According to interRAI** | **Descriptive Analysis** | **Inferential Analysis** |
| --- | --- | --- | --- |
| 1 | Private Home/Apartment/Rented Room | Private Home | Private Home |
| 2 | Board and Care | Assisted Board | Other |
| 3 | Assisted Living or Semi-independent Living | Assisted Board | Other |
| 4 | Mental Health Residence | LTC/Hospital/Died | Other |
| 5 | Group Home for Persons with Physical Disability | Assisted Board | Other |
| 6 | Setting for Persons with Intellectual Disability | Assisted Board | Other |
| 7 | Psychiatric Hospital/Unit | LTC/Hospital/Died | Other |
| 8 | Homeless (with or without shelter) | LTC/Hospital/Died | Other |
| 9 | Residential Care Facility (e.g., Long-term Care Home, Nursing Home) | LTC/Hospital/Died | Other |
| 10 | Rehabilitation Hospital/Unit | LTC/Hospital/Died | Other |
| 11 | Hospice facility/Palliative Care Unit | LTC/Hospital/Died | Other |
| 12 | Acute Care Hospital/Unit (Return Not Expected) | LTC/Hospital/Died | Other |
| 13 | Correctional Facility | LTC/Hospital/Died | Other |
| 14 | Continuing Care Hospital/Unit | LTC/Hospital/Died | Other |
| 15 | Other | LTC/Hospital/Died | Other |
| 16 | Deceased | LTC/Hospital/Died | Other |
| 17 | Acute Care Hospital (Return Expected) | LTC/Hospital/Died | Other |
