# Supplementary figures and images for: Multistate Competing Risk Analysis of Transition Back to the Community Among Long-Term Care Home (LTC) Destined Patients: A Brief Report
Source: J Prim Care Community Health. 2023 Dec 22;14:21501319231220742. doi: 10.1177/21501319231220742 (PMC10748573; doi:10.1177/21501319231220742)

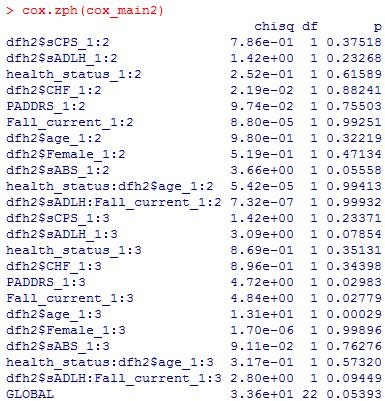

Supplement: sj-docx-2-jpc-10.1177_21501319231220742 – Supplemental material for Multistate Competing Risk Analysis of Transition Back to the Community Among Long-Term Care Home (LTC) Destined Patients: A Brief Report [file sj-docx-2-jpc-10.1177_21501319231220742.docx]
